# Comparative Analysis of XFEM and Phase Field Approaches for Fracture Prediction in Flexible Ti-6Al-4V Thoracic Implants

**DOI:** 10.3390/jfb17050222

**Published:** 2026-05-02

**Authors:** Alejandro Bolaños, Alejandro Yánez, Alberto Cuadrado, María Paula Fiorucci

**Affiliations:** Biomaterials and Biomechanics Research Group, Department of Mechanical Engineering, University of Las Palmas de Gran Canaria, 35017 Las Palmas of Gran Canaria, Spain; alejandro.yanez@ulpgc.es (A.Y.); alberto.cuadrado@ulpgc.es (A.C.); paula.fiorucci@ulpgc.es (M.P.F.)

**Keywords:** phase field (PF), extended finite element method (XFEM), thoracic implant, cohesive zone model (CZM), fracture model

## Abstract

The scientific literature increasingly supports the use of computational models to predict fracture across a wide range of applications, which, when calibrated with experimental data, can yield highly consistent results. Although the extended finite element method (XFEM) is widely used in commercial packages, phase field (PF) methods have emerged as a robust alternative. In this study, a cohesive zone model (CZM) was implemented using both approaches (a PF model with an implicit damage initiation criterion and a standard commercial XFEM solver with an explicit damage initiation criterion) to analyze their robustness and computational efficiency. First, a standardized fracture test of a compact tension (CT) specimen was simulated and compared with experimental data to validate both methods, achieving accurate predictions under plane strain conditions with a dominant mode I fracture behavior. Subsequently, the application of both fracture models was extended to flexible thoracic prostheses across two distinct chest wall reconstruction scenarios: a single-rib unilateral model and a multi-rib bilateral configuration. An extreme-case compressive displacement was assessed to identify critical regions susceptible to fracture initiation and to evaluate the structural limits of the proposed designs. The results showed that the PF approach required a higher computational time, but exhibited more stable convergence. In contrast, the XFEM-based solver required careful mesh calibration to ensure convergence under complex conditions. These results highlight the potential of the PF approach as a practical tool for identifying and improving critical regions of implants, overcoming the limitations of commercial XFEM implementations.

## 1. Introduction

In recent years, personalized medicine has undergone remarkable growth driven by advances in manufacturing technologies that have enabled the production of patient-specific implants with highly complex geometries and a wide range of materials. In the field of thoracic surgery, powder bed fusion (PBF) techniques have facilitated the development of flexible thoracic implants for chest wall reconstruction made from biocompatible metallic alloys [1,2]. These implants represent a promising alternative to the rigid meshes and plates still commonly used in these procedures, which result in a high percentage of postoperative complications [3].

A thorough investigation of these innovative designs is essential, as their irregular and intricate geometries, which confer enhanced flexibility (hyper-flexibility), cause them to undergo large deformations within the human thorax. Considering the various mechanisms that can lead to the failure of additively manufactured implants, such as fatigue induced by cyclic in vivo loading [4,5,6], or fracture resulting from small defects introduced during the manufacturing process [7], it is essential to conduct comprehensive computational analyses prior to implantation. Such preliminary studies are critical to validate the mechanical performance of the designs and ensure their long-term structural integrity and safety before clinical application.

Due to the novelty of these designs, the current available literature in the field of thoracic prothesis is mainly limited to basic computational analyses of stress and strain under quasi-static loading conditions [8,9,10,11]. Therefore, further computational research employing advanced numerical methods is required to accurately assess the fracture mechanisms and structural failure behavior of these implants. For this purpose, the computational approaches used in this area have evolved considerably in recent years. Of these, the finite element method (FEM) has long been the standard tool, but its ability to model crack propagation accurately and efficiently remains limited. These limitations do not arise solely from the presence of crack-tip singularities, but more fundamentally from the continuous nature of standard finite element formulations, which are unable to represent displacement discontinuities across crack surfaces [12]. As a consequence, classical FEM requires either remeshing during crack growth or the alignment of the mesh with the expected crack path, which increases the computational cost and may introduce numerical inaccuracies, particularly when crack trajectories are unknown or complex [13,14]. To overcome these limitations, advanced techniques such as the extended FEM (XFEM) and phase field (PF) methods have been developed, each based on fundamentally different theoretical principles [12].

XFEM extends the classical FEM approximation space through the partition of unity enrichment, enabling the incorporation of discontinuous functions and near-tip asymptotic fields without requiring mesh conformity to the crack geometry. This allows cracks to initiate and propagate independently of the mesh, significantly improving efficiency in problems involving the evolution of discontinuities [14]. Due to these advantages, XFEM has been widely integrated into commercial software packages [15], proving particularly effective in the fracture analysis of heterogeneous materials and complex geometries, with extensive applications in the design of orthopedic and dental implants [16,17,18]. Its main advantage lies in its ability to accurately capture the stress-field singularity at the crack tip, making it well-suited for predicting quasi-brittle fracture, a common failure mode in some biomedical materials [13,19]. In contrast, phase field (PF) methods rely on a variational formulation of fracture in which cracks are represented as diffused damage regions governed by a continuous scalar field. Crack evolution is driven by energy minimization principles and controlled by an internal length scale parameter, which regularizes the sharp crack topology. This framework naturally captures complex phenomena such as crack nucleation, branching, and coalescence without the need for explicit crack tracking, although it typically requires finer discretization and higher computational cost [12]. The PF approach has gained significant attention in recent years due to its ability to predict complex fracture paths without prior assumptions about the propagation direction [20,21]. Its thermodynamic foundation makes it particularly suitable for modeling complex fracture phenomena, such as those occurring in viscoelastic materials or under fatigue damage, positioning it as a promising research field in biomechanics [22,23,24].

The present work continues previous research on the feasibility of a novel flexible thoracic implant design based on a spring-like geometry. The first of these studies consisted of the biomechanical evaluation, both experimental and computational, of two initially proposed geometries [25]. The chosen design was also implemented in a 2018 clinical case, in which the fracture of a bilateral implant occurred, leading to the redesign of the most critical sections identified as fracture-prone [26]. Subsequently, a second study was conducted to further analyze the improved design. Several isolated rib levels were examined, and multiple implant configurations based on this new design were included in a complete 3D thoracic cage model. This study aimed to achieve a more realistic understanding of the implant behavior under quasi-static conditions by analyzing stress and strain distributions during unfavorable sternum loading scenarios, such as the application of a cardiopulmonary resuscitation maneuver [11].

Nevertheless, while most existing computational evaluations of thoracic implants are limited to analyzing stress and strain distributions [8,10,27], the application and comparative performance of different fracture modeling strategies in highly deformable, patient-specific thoracic implants has not been systematically studied in the literature. To address this gap, fracture prediction was based on a cohesive zone model (CZM) that was implemented using two distinct computational frameworks: a PF (PF-CZM) approach and the standard commercial implementation of XFEM (XFEM-CZM) in Abaqus (Dassault Systèmes, SIMULIA Corp., Providence, RI, USA). In both approaches, the cohesive formulation provides a common energy-based framework that governs damage initiation and evolution through a traction–separation relationship, enabling a unified description of fracture energy dissipation across the two methodologies [28]. The PF model was defined with an implicit damage initiation criterion, whereas the built-in commercial XFEM solver was configured with an explicit damage initiation criterion that additionally governs the crack propagation direction. This distinction, while sharing a common cohesive energy-based framework, enables the assessment of how different crack initiation and propagation mechanisms influence fracture predictions in geometrically complex biomedical structures subjected to multiaxial loading conditions.

As an initial validation step, standardized fracture-toughness tests were simulated using compact tension (CT) specimens made of Ti-6Al-4V extra-low interstitial (ELI) alloy manufactured using LPBF technology. The simulations were conducted to validate both fracture models by comparing their results with the experimental data and reference material parameters available in the literature. Once validated under plane strain conditions, both models were applied to simulate crack propagation in two implant configurations based on the proposed hyper-flexible design, intended to reconstruct different rib levels. The application of both fracture models enabled the identification of critical implant regions most susceptible to failure and provided a comprehensive comparative assessment of their predictive capabilities. This evaluation encompassed both quantitative and qualitative aspects, including crack path evolution, numerical robustness, and sensitivity. By focusing on fracture-driven failure under quasi-static loading, this work provides a critical assessment of the designs’ mechanical performance, ensuring both structural integrity and safety ahead of clinical implementation.

## 2. Materials and Methods

### 2.1. Computational Analysis of Experimental Testing

**Figure 1:**
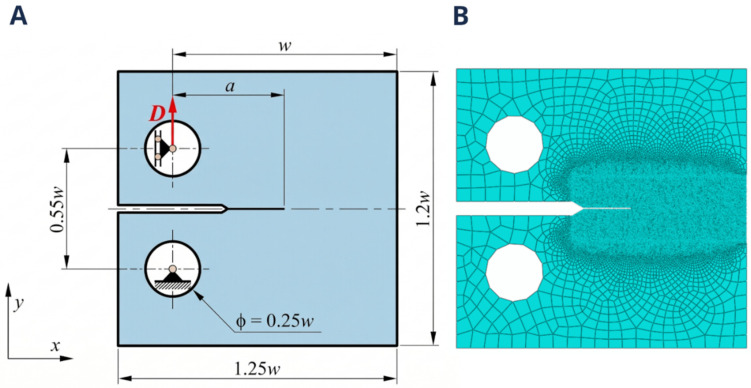
(**A**) Compact tension (CT) specimen with the dimensions and boundary conditions applied in the finite element (FE) software (version: Abaqus 6.14). (**B**) Mesh of the model with a maximum refinement of 0.05 mm in the fracture region.

The computational simulations to study fracture in the CT specimens were performed using Abaqus (version: Abaqus 6.14) with a specimen matching the dimensions: *B* = 20 mm, *w* = 50 mm, *a*/*W* = 0.5 and *f*(*a*/*w*) = 9.67, with the geometry defined to comply with the specifications of ASTM E1820-08a [29]. In this context, the parameter *a* represents the initial crack length, defined as the distance from the displacement line to the tip of the pre-crack, while *w* denotes the characteristic width of the specimen and *B* is the corresponding thickness. Given this specimen thickness, the simulation was carried out under plane strain conditions, with a nominal width of 20 mm specified for force scaling. All specimen measurements are described in Figure 1A.

The Ti-6Al-4V alloy assigned was modeled with a Young’s modulus (*E*) of *E* = 110.4 GPa and a Poisson’s ratio (*v*) with a value of *v* = 0.3 [30]. Although a linear elastic constitutive model was adopted for the bulk material, fracture behavior was governed by a cohesive zone model (CZM), which introduces progressive stiffness degradation and energy dissipation within a finite process zone (see Section 2.3 and Section 2.4). This approach allows the effective representation of fracture mechanisms, capturing the macroscopic effects of inelastic phenomena such as plasticity without explicitly defining a plastic constitutive law. All degrees of freedom of the nodes located on the circumferential surfaces defining the upper and lower pin holes were coupled to reference points located at their respective centers. For the lower pin, both horizontal (*x*-axis) and vertical (*y*-axis) displacements were constrained; for the upper pin; only the horizontal displacement (*x*-axis) was constrained, allowing free rotation in both cases. A displacement of 3 mm was then applied in the positive *y*-direction at the upper pin to open and fracture the CT specimen, at an equivalent loading rate of 1 kN/s.

Crack propagation was analyzed using both the PF-CZM and XFEM-CZM approaches, whose detailed configurations are provided in Section 2.3 and Section 2.4, respectively. In the PF-CZM, CPE_4_T elements (4-node plane strain, thermally coupled quadrilateral, bilinear displacement, and temperature) were used. For the XFEM-CZM, CPE_4_R elements (4-node bilinear plane strain quadrilateral, reduced integration, hourglass control) were employed, with a partitioning strategy allowing gradual mesh refinement across the specimen in both cases. The element size in the fracture region was reduced to 0.05 mm (Figure 1B).

### 2.2. Obtention and Configuration of Thoracic Implant Models in Finite Element (FE) Software

To simulate thoracic reconstruction, a 3D model of a healthy ribcage was obtained from a computed tomography scan of an adult patient. This model was then used to study the designs of various flexible prostheses under fracture conditions. The thorax was scanned using a clinical computed tomography scanner (VCT 64 c/s, General Electric, Chicago, IL, USA) with a slice thickness of 0.6 mm and a slice interval of 0.6 mm, and the segmentation was performed using a density threshold to exclusively identify the bony structures of the thorax. A parametric model representing the ribs with a uniform cortical thickness of 0.75 mm was then generated [31]. The 3D model was subjected to mesh cleaning to ensure compatibility and was finally exported in Parasolid format and imported into Abaqus software for the analysis. Further details of this model can be found in a previous work [11].

**Figure 2:**
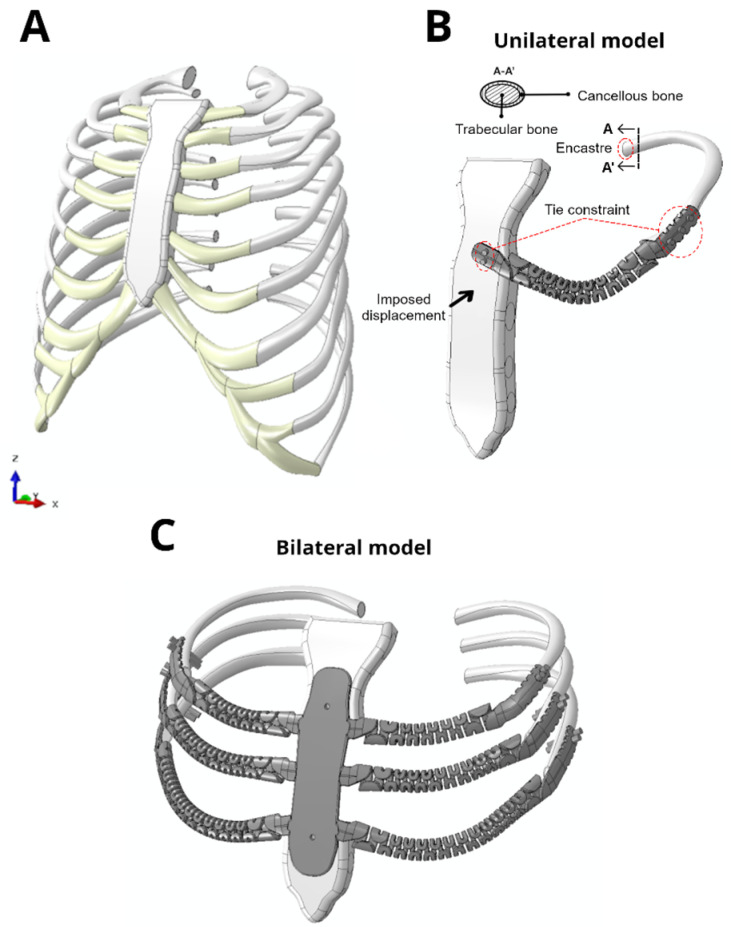
(**A**) Complete model of the healthy thoracic cage obtained from computed topography images. (**B**) Model of the left third rib level reconstructed with a flexible implant. (**C**) Model of the third, fourth, and fifth rib levels reconstructed with a bilateral flexible implant. Models B and C were obtained by resection and modification of the complete model A.

Once the complete 3D thoracic model was obtained (Figure 2A), two reconstruction models were derived and subsequently analyzed in terms of fracture: one using the XFEM-CZM available in Abaqus, and the other employing the PF-CZM. First, a unilateral model was developed by isolating the left third rib to simulate its reconstruction with an implant, along with the sternum (Figure 2B). Second, a bilateral model was reconstructed, consisting of the third, fourth, and fifth rib levels together with the corresponding section of the sternum (Figure 2C). In this case, the rib implants were connected to a central fixation plate attached to the remaining portions of the sternum, which served as a common anchoring structure for the implants. This additional configuration allows for the analysis of multi-level reconstructions under more anatomically representative conditions. The configurations had to be simplified due to the high computational cost of PF simulations. This limitation becomes particularly critical in models with a large number of elements, where the computational cost rapidly increases, especially because a very fine mesh refinement is required in the vicinity of the crack tip to accurately capture the evolution of the damage field [12]. Furthermore, the choice of these rib levels was based on the exclusion of the second level, whose implant exhibited excessive stiffness in previous studies [11], attributed to the distinct biomechanical behavior of the first two rib levels [32]. After obtaining the final models (Figure 2B,C), tie constraints were defined between the screws of the sternum and the thoracic prosthesis, as well as between the cortical and trabecular bone tissue and the implant, ensuring the correct coupling of both models.

A boundary condition was also applied to the posterior end of the ribs by imposing an encastre, nullifying all rotations and displacements in both models. Considering that these are isolated models, the pump handle angle and bucket handle angle generated in the ribs are considered negligible because the sternum is not subjected to the other forces produced by the remaining ribs of the thoracic cage in normal conditions. For this reason, and also considering the low caliper angle (experienced around the *z*-axis) due to the small displacements that the sternum undergoes in the laterolateral direction [33], all angles and displacements at the posterior end were restricted to simplify the boundary conditions. Also, a maximum compression displacement of 100 mm in the anteroposterior direction (along the *y*-axis) was applied in the central region of the sternum for the unilateral model and in the central region of the prosthetic plate for the bilateral model, simultaneously limiting displacements in any other direction to force the fracture of the implant in a pure compression movement. Since a compression displacement of 50 mm corresponds to a 50% fracture probability [34], it is reasonable to assume that a displacement of 100 mm would almost certainly lead to rib failure before fracture occurs in the implant. Nevertheless, this displacement magnitude was intentionally selected in order to specifically explore the fracture limits of the prosthesis itself, independently of the surrounding bone structures. It should be noted that this loading condition exceeds the physiological thoracic compression levels, which are typically limited to approximately 50–60 mm during cardiopulmonary resuscitation [35]. Therefore, the applied displacement should be interpreted as an extreme-case computational fracture test rather than a physiological scenario. Its aim is to identify critical regions and support the assessment of the hyper-flexible response of the proposed design, which is intended to sustain large deformations without undergoing premature fracture.

Finally, the material properties of all human tissues in both models were considered under a linear elastic isotropic model. Although bone tissue exhibits anisotropic and, in some cases, nonlinear mechanical behavior, this simplification is commonly adopted in thoracic finite element models to reduce computational cost while still providing an accurate representation of the global structural response. This modeling strategy was previously validated by the authors [11], where a full ribcage model based on these simplified properties showed strong agreement with more complex models.

A value of *E* = 14.4 GPa was assigned to the cortical bone, while *E* = 40 MPa was used for the trabecular bone [36]. The corresponding Poisson’s ratios were *v* = 0.3 and *v* = 0.35, respectively [37]. This assumption may affect local stress distributions within the bone; however, its influence on the present study was limited, as the primary objective was the evaluation of implant fracture rather than bone failure or implant–bone interaction effects. Also, the material properties assigned to the Ti-6Al-4V implants were defined according to the characteristics described in the previous section. All finite element simulations performed in this study were conducted on a workstation equipped with an Intel Core i9-11900K processor and 32 GB of RAM (Intel, Santa Clara, CA, USA).

### 2.3. Fracture Modeling Using the Extended Finite Element Method (XFEM)

For fracture prediction in all models using XFEM-CZM, including the CT specimen as well as the unilateral and bilateral implant models, an explicit damage criterion based on a cohesive traction–separation law was adopted. Within this approach, the cohesive zone model is embedded into the XFEM formulation to provide a physically based description of damage initiation and evolution through a well-defined traction–separation relationship. This is particularly powerful for simulating crack nucleation and propagation without requiring the mesh to conform to the discontinuity [14]. In addition, the cohesive formulation contributes to a more controlled and numerically stable representation of fracture, as it regularizes the damage process and ensures a consistent energy dissipation mechanism during crack growth. This model of fracture is currently applied to a wide range of materials such as metals, polymers, ceramics, or composites [28,38], and has even been used in the machinability processes of Ti-6Al-4V [39]. It also enables fracture prediction in structures without pre-existing cracks, a major limitation of linear elastic fracture mechanics (LEFM)-based fracture models [40].

The theoretical foundation of XFEM is based on the enrichment of the classical finite element approximation through the partition of the unity method, which allows the displacement field to represent discontinuities independently of the mesh topology. In this framework, the enriched displacement field can be described as:

(1) $$u=\;\sum_{j=1}^{n}N_{j}\left(x\right)\left[u_{j}+H\left(x\right)a_{j}+\sum_{\alpha =1}^{n_{e}\left(j\right)}F_{\alpha }\left(x\right)b_{j}^{\alpha }\right]$$

In Equation (1) *N*_*j*_(*x*) are the standard finite element shape functions and *u_j_* are the nodal displacements. The function *H*(*x*) is the Heaviside function representing the displacement discontinuity across the crack surfaces (Equation (2)), while *F_α_*(*x*) are the asymptotic near-tip enrichment functions that reproduce the stress singularity at the crack front. The additional degrees of freedom *a_j_* and $b_{j}^{\alpha }$ correspond to the enriched nodes, and *n_e_*(*j*) denotes the number of enrichment functions associated with node *j.* This approximation introduces additional degrees of freedom only in nodes affected by the discontinuity.

(2) $$H\left(x\right)=\left\{\begin{array}{c} 1,\;if\left(x-x^{*}\right)n\geq 0\\ -1,\;\mathrm{otherwise} \end{array}\right.$$

where *x* is the arbitrary point near the crack face, and x* is the closest point to x, which is located on the crack surface. *n* is the outward unit vector normal to the crack at point x*.

Damage initiation is governed by the maximum principal stress (*MAXPS*) criterion, whereby crack nucleation occurs once the maximum principal stress at a material point reaches a predefined threshold strength, σ^0^_max_, according to Equation (3). In this case, the damage initiation function is expressed as:

(3) $$f=\frac{\left\{\sigma_{max}\right\}}{\sigma_{max}^{0}}$$

In Equation (3), $\sigma_{max}^{0}$ denotes the maximum principal stress, which in this case was assigned a value of 1150.88 MPa, corresponding to the measured ultimate tensile strength of the material according to experimental tests [30]. The symbol { } represents the Macaulay brackets, indicating that compressive stresses were not considered for damage initiation. Also, a tolerance of 0.05 was employed for the *MAXPS* damage initiation criterion. This means that the damage function *f* is considered satisfied when it slightly exceeds the threshold, specifically within the range 1 < *f* < 1.05. Abaqus allows this small overshoot for numerical reasons, and subsequently adjusts the increment so that damage initiation occurs precisely at *f* = 1.

**Figure 3:**
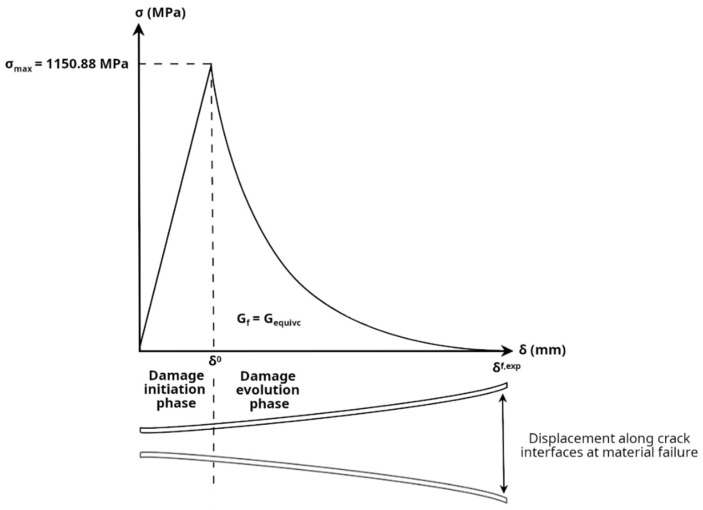
Damage model implemented in the finite element (FE) software, relating to the maximum principal.

Once this criterion is met, a new crack is introduced into the computational domain, oriented orthogonal to the maximum principal stress direction. This approach has been employed in the literature to define crack propagation paths, including in fatigue and quasi-static analyses of biocompatible metal alloys [18,41,42]. The subsequent propagation of this crack is governed by an energy-based law, as the combination of a stress-based initiation criterion and an energy-based propagation criterion can provide a robust and physically sound description of material failure. Specifically, material degradation is controlled by the fracture energy of the material (*G_f_*), which corresponds to the total area under the traction–separation law curve [43] (Figure 3). To determine the fracture energy, the mode I fracture toughness (*K_Ic_*) is required. Considering the manufacturing process of the Ti-6Al-4V alloy used for the implants and the post-heat treatment applied consisting of annealing at 850 °C [30], the mode I fracture toughness value was then adopted from the literature under an annealed condition at 800 °C, corresponding to *K_Ic_* = 60 MPa⋅$\sqrt{mm}$ [44]. Since the annealing heat treatment below the *β* transus temperature typically promotes the formation of coarser *α* lamellae without influencing the size or shape of the *β* phase [45], the material was analyzed as quasi-brittle, and the use of this value was considered a conservative assumption. Furthermore, the corresponding critical energy release rate in mode I (*G_Ic_*) was calculated under plane strain conditions according to Equation (4), resulting in *G_Ic_* = 29.67 N/mm.

(4) $$G_{Ic}=\frac{K_{IC}^{2}\;∙\;(1-v^{2})}{E}$$

Therefore, *G_f_* was approximated by the critical energy release rate (*G_equivC_*). To compute it, a mixed-mode formulation based on the power law criterion proposed by Wu and Reuter [46] was implemented in all models. This criterion is expressed in Equation (5) and has been widely used in the literature, especially for fracture toughness standardized tests [42,47].

(5) $$\left(\frac{G_{equiv}}{G_{equivC}}\right)={\left(\frac{G_{I}}{G_{IC}}\right)}^{a_{m}}+{\left(\frac{G_{II}}{G_{IIC}}\right)}^{a_{n}}+{\left(\frac{G_{I}}{G_{IIIC}}\right)}^{a_{0}}$$

In Equation (5), *G_Ic_*, *G_IIc_*, and *G_IIIc_* correspond to the mode I, II, and III critical energy release rates of the material, all of which were taken as 29.67 N/mm with all exponents set to 1 for simplicity. Finally, an exponential softening law was applied in the damage curve (Figure 3) after reaching the strength limit, in order to simulate sudden material degradation—a typical behavior of materials exhibiting quasi-brittle fracture, such as Ti-6Al-4V under specific conditions [48], particularly in regions of stress concentration, as also reported in previous experimental studies [25]. In the Abaqus implementation, the software itself adjusts the curvature of this exponential function through a parameter *α*, ensuring that the area under the complete curve matches the specified fracture energy as described in Equation (6).

(6) $$\delta^{f,exp}=\frac{G_{f}\cdot (e^{\alpha }-1)\cdot \alpha }{\sigma_{max}\cdot (e^{\alpha }-\alpha -1)}$$

In Equation (6), *δ*^*f*, *exp*^ is the distance between the crack interfaces at the maximum damage, expressed as a function of the parameter *α,* the material’s fracture energy (*G_f_*), and the maximum principal stress (*σ_max_*).

Finally, the CT specimen was meshed as described in Section 2.1, while both the unilateral and bilateral implant models were meshed with 3D stress elements of type C_3_D_10_ (10-node quadratic tetrahedron) across all assembly components. The implant was defined as the crack growth domain, with enriched elements implemented through the XFEM module. A global element size of 3 mm was used for all structures, while a finer mesh of 0.75 mm was applied to the implants in both models. To ensure the objectivity and mesh-independence of the numerical results, a convergence study was designed for the expected crack initiation region, identified from preliminary simulations. This analysis evaluated progressive local refinements using element sizes of 0.50 mm, 0.30 mm, and 0.20 mm to determine the optimal discretization required to accurately capture the critical failure load.

### 2.4. Fracture Modeling Using the Phase Field (PF) Approach

Fracture prediction in all models was also carried out using the PF approach, which is widely employed to simulate crack initiation and propagation in materials through the introduction of a continuous scalar damage variable, denoted as *φ*. This regularization function smoothly varies from 0, representing the intact material, to 1, corresponding to fully fractured material, thereby describing the transition between damaged and undamaged regions within a diffuse fracture zone. By avoiding the need to explicitly track sharp crack surfaces, the PF approach provides a robust framework for capturing complex fracture phenomena, including crack branching and coalescence. Nevertheless, one of its main limitations lies in its high computational cost, as obtaining accurate results requires fine mesh refinement in the vicinity of the evolving fracture zone, which substantially increases the numerical effort [12].

The phase field formulation of fracture is based on a variational approach in which crack evolution is governed by the minimization of the total energy of the system. In this framework, the fracture problem is defined through the following regularized energy functional:

(7) $$\Psi =\int_{\Omega }\left[{\left(1-\emptyset \right)}^{2}\psi_{0}\left(\varepsilon \right)+G_{c}\left(\frac{\emptyset^{2}}{2l}+\frac{l}{2}{\left|\nabla \emptyset \right|}^{2}\right)\right]d\Omega$$

In Equation (7) the parameter *Ψ*_0_ (*ε*) represents the strain energy density of the undamaged material, *G_c_* is the critical energy release rate, and *ℓ* is a length scale parameter that governs the size of the fracture process zone. The variable ∅ represents the phase field, which regularizes the sharp crack topology by approximating it as a continuous damage distribution. The governing equations are obtained from the minimization of this functional, resulting in a coupled system consisting of the mechanical equilibrium equation and the phase field evolution equation. This formulation enables the natural simulation of crack initiation, propagation, and complex crack patterns without the need for explicit crack tracking.

In this study, the UMAT and HETVAL subroutines proposed by Navidtehrani et al. [49,50] were implemented in Abaqus using the PF-CZM constitutive model to simulate a quasi-brittle material response, incorporating the exponential softening law described by Wu et al. [51]. Within this framework, the cohesive zone model is employed to define an explicit traction–separation relationship that governs the fracture process, ensuring a consistent and physically interpretable description of energy dissipation during crack initiation and growth. This cohesive formulation is fully coupled with the phase-field description, allowing the fracture process to be represented in an energy-consistent manner while preserving numerical robustness and facilitating comparison with the XFEM-CZM approach. According to the PF-CZM model, the degradation function used follows Equation (8):

(8) $$g\left(\emptyset \right)=\frac{{\left(1-\emptyset \right)}^{d}}{{\left(1-\emptyset \right)}^{d}+a\emptyset (1+b\emptyset )}$$

where the parameter *a* depends on the mechanical properties of the material, while *b* and *d* are constants with values *b* = 2^(5/3)^–3 and *d* = 2.5 for the exponential model [49].

Furthermore, to prevent material degradation under compressive states, an energy split following the approach proposed by Amor et al. [52] was adopted. This criterion is based on the spectral decomposition of the strain tensor, ensuring that only the elastic energy associated with tensile stresses is degraded, similarly to the *MAXPS* criterion in terms of stresses, which considers only tensile stresses for damage initiation. Also, a staggered solution scheme was employed in order to provide a more robust and flexible solver under quasi-static conditions.

In the subroutine, in addition to Young’s modulus and Poisson’s ratio, two key parameters must be introduced: the critical energy release rate (*G_c_*) and the characteristic length of the diffuse crack width (*ℓ*), considering the expression of the parameter *a* in Equation (9).

(9) $$a=\frac{4EG_{c}}{\pi l\sigma_{f}^{2}}$$

This constant is included in the quadratic degradation function *g*(*φ*), which governs material degradation in this model. The *G_c_* used was 29.67 N/mm, as determined previously. Regarding parameter *ℓ*, which defines the width of the diffuse damage zone, it is intrinsically related to the mechanical properties of the material in the Ambrosio–Tortorelli (AT1 and AT2) constitutive models [53]. This differs from the PF-CZM, which is directly related to the material’s fracture stress *σ_f_*. In the present study, the parameter *ℓ* was set to 0.30 mm, and the fracture stress was approximated by the material’s ultimate tensile strength, with a value of 1150.88 MPa [30]. For further information on the routines, please refer to the author’s documentation.

**Figure 4:**
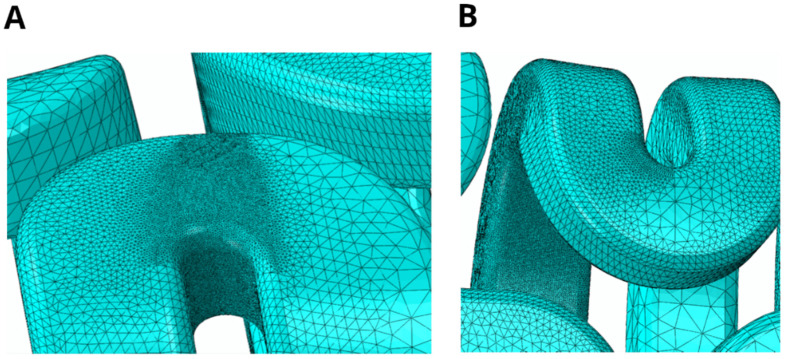
(**A**) Mesh refinement in a critical area located in the unilateral implant. (**B**) Mesh refinement in a critical area located in the bilateral implant. In both cases, a minimum element size of 0.05 mm was used in the areas where damage was expected.

According to the literature, it is important to note that within the fracture zone, the mesh element size (*h*) should be significantly smaller than *ℓ*. Although it is generally recommended to follow the relation *h* ≤ *ℓ*/2 [54] to minimize mesh discretization effects, recent studies suggest an even more restrictive criterion, *h* ≤ *ℓ*/5, in the fracture zone under shear stresses [55]. Consequently, this more restrictive requirement was adopted. To satisfy this requirement, all implant models were meshed using 3D stress elements of type C_3_D_10_ for all assembly components, except for the implant. In this case, C_3_D_10_MT elements (10-node, modified, thermally coupled, tetrahedral) were employed. Prior to the final simulations, a mesh convergence study was performed considering element sizes of 0.20 mm, 0.10 mm, and 0.05 mm in the fracture process zone to ensure that the results were independent of the discretization. A global element size of 3 mm was adopted for all structures, while a finer mesh of 0.75 mm was applied in the implants of both models, with additional refinement down to 0.05 mm in the critical regions—as shown in Figure 4—achieving an *ℓ*/*h* ratio of 6.

Bourdin et al. [56] pointed out that mesh discretization in FE models leads to an increase in the fracture energy. Therefore, *G_c_* was adjusted in the simulations by considering the parameters *h* and *ℓ* in order to obtain the corrected value $G_{c}^{cor}$ through Equation (10):

(10) $$G_{c}^{cor}=\frac{G_{c}}{1+\frac{h}{2l}}$$

Finally, it is important to note that in the case of the CT specimen model described in Section 2.1, in addition to defining the pre-crack geometrically, a temperature boundary condition was applied on the edges defining the notch contour, with a value set very close to 1. According to the work of Infante et al. [55], this approach better captures the energy of the model. In the remaining models, both the unilateral and bilateral ones, no temperature boundary condition was imposed, since no pre-crack was present, thereby allowing the model itself to determine the diffuse damage zones for fracture predictions.

## 3. Results and Discussion

### 3.1. Standardized Fracture Toughness Test

**Figure 5:**
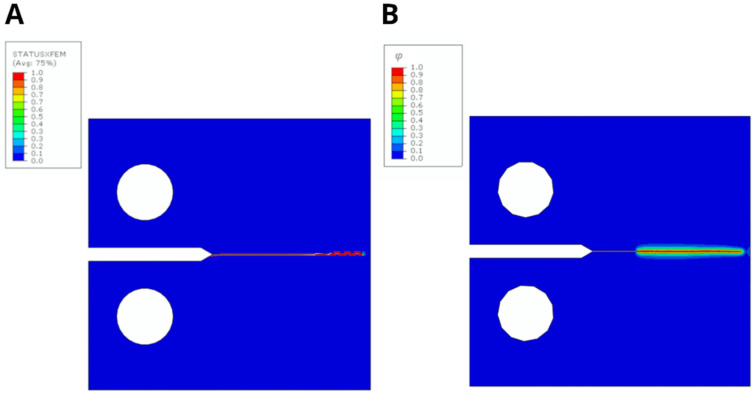
(**A**). Fracture prediction obtained with XFEM-CZM showing the STATUS XFEM damage variable: values of 0 indicate that the material is intact, while values of 1 correspond to fully damaged regions. (**B**). Fracture prediction obtained with PF-CZM as a function of the damage variable φ, with the same meaning.

The fracture simulation results of the CT specimens are shown in Figure 5 for both the XFEM-CZM and PF-CZM, illustrating material damage through the output variables *STATUS XFEM* and the PF function *φ*, respectively, with values ranging from 0 to 1 according to material damage.

**Figure 6:**
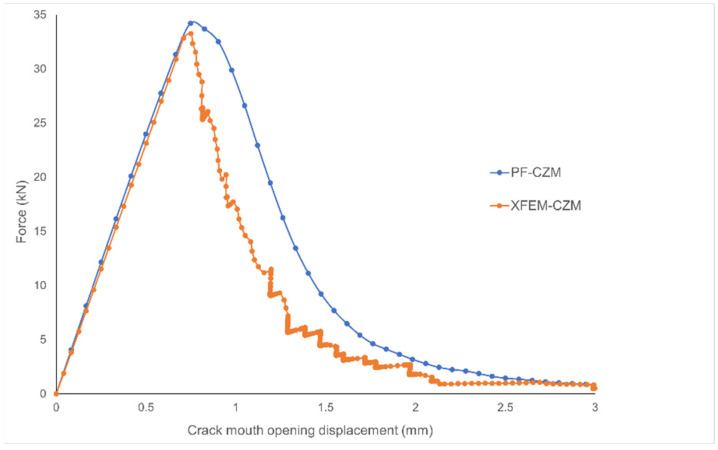
Force–displacement curve obtained for the CT specimen using the phase field (PF) method and the extended finite element method (XFEM).

In both cases, a completely straight fracture path was observed, as expected due to the dominant mode I fracture under plane strain conditions. PF-CZM produced a diffuse, straight damage zone, while XFEM-CZM predicted a slight upward crack deviation that started approximately 20 mm from the notch tip. Under the applied opening displacement, in the PF-CZM, the crack propagated until it was about 0.84 mm away from the specimen edge, whereas in the XFEM-CZM, this distance was 1.34 mm. The visual differences in the fracture mode observed in both cases were minimal. Figure 6 also shows the force–displacement curve obtained in each case, with the displacement represented as the crack mouth opening displacement (CMOD).

The initial elastic region and the fracture point occurred at nearly the same CMOD in both cases, with maximum loads of 33.25 kN for XFEM-CZM and 34.19 kN for PF-CZM, corresponding to a symmetric relative difference of 2.79% and a displacement at fracture of 0.74 mm in both cases. In comparison with the experimental results, Sedmak et al. [16] conducted tests on CT specimens made of the same material, with a thickness of *B* = 25 mm. They reported an average maximum load per unit thickness of 1.65 kN/mm, whereas the values obtained were 1.66 kN/mm for the XFEM-CZM model and 1.70 kN/mm for the PF-CZM model. Therefore, given the minimal difference obtained in both approaches in comparison to the experimental data, the results were considered consistent. Beyond the fracture point, a smoother softening behavior was observed for the implemented PF-CZM model, whereas XFEM-CZM exhibited a more abrupt and irregular softening response. This can be attributed to the localized nature of damage initiation and propagation in cohesive formulations with an explicit criterion to initiate the damage, where crack evolution is governed by discrete failure parameters. In contrast, PF-CZM regularizes the crack growth process over a diffusive damage zone, leading to smoother and more gradual degradation of stiffness. Consequently, while XFEM-CZM captured the onset of fracture more sharply, it inherently produced a less stable post-peak response than the PF framework.

### 3.2. Unilateral and Bilateral Models

#### 3.2.1. Mesh Sensitivity and Computational Efficiency

**Figure 7:**
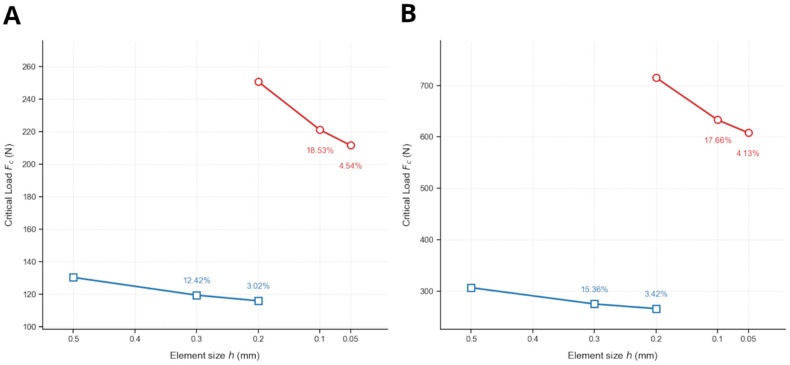
Mesh convergence analysis for (**A**) unilateral and (**B**) bilateral thoracic models. Relative errors are indicated for each refinement level, calculated relative to the final value at maximum refinement (decreasing element size h: Mesh Refinement). Values below 5% indicate that the critical load Fc has reached a stable mesh-independent solution for both numerical frameworks. (XFEM-CZM curves in blue and PF-CZM curves in red).

To ensure the objectivity of the numerical predictions, a mesh sensitivity analysis was performed with an assessment of the computational performance for both the XFEM-CZM and PF-CZM frameworks. The critical failure load *F_c_* was monitored across three levels of refinement, as illustrated in Figure 7. This force was defined as the peak load of the force–displacement curves for each model.

As observed in Figure 7, the XFEM-CZM models demonstrated a relatively low sensitivity to mesh size for the prediction of *F_c_*. For the unilateral model (Figure 7A), the relative error decreased from 12.42% to 3.02% for element sizes of *h* = 0.5 mm and *h* = 0.3 mm, respectively, relative to the finest mesh of *h* = 0.2 mm. Similarly, the bilateral model (Figure 7B) showed a variation of 15.36% for the coarsest mesh, dropping to 3.42% at *h* = 0.3 mm. In contrast, the PF-CZM models exhibited a significantly higher sensitivity to the element size. For the unilateral case, the relative error decreased from 18.53% to 4.54% for element sizes of *h* = 0.2 mm and *h* = 0.1 mm, respectively, relative to the finest mesh of *h* = 0.05 mm. The bilateral model followed a similar trend, with relative errors of 17.66% and 4.13% for the same mesh sizes, respectively, before reaching convergence at *h* = 0.05 mm. This behavior is consistent with the intrinsic characteristics of the phase field formulation, in which the accuracy of the solution is governed by the ratio between the internal length scale (*ℓ*) and the element size (*h*). In particular, an adequate resolution of the diffused fracture zone requires *ℓ*/*h* ≥ 5 [55]. In the present study, only the finest mesh satisfied this condition (*ℓ*/*h* ≈ 6 for *h* = 0.05 mm), whereas coarser meshes did not, which explains the higher discrepancies observed for larger element sizes.

Furthermore, it is worth noting the convergence behavior of both methods. While XFEM-CZM reached mesh independence with fewer elements, it frequently exhibited numerical instabilities. XFEM-CZM involves nominally three degrees of freedom (DOFs) per node (excluding enrichment contributions) and strongly couples the displacement field with the cohesive traction–separation law along the discontinuity. This makes the solution sensitive to mesh size and element distortion. Moreover, the enrichment strategy and the need for accurate numerical integration over discontinuities further increase this sensitivity. On the other hand, the staggered solution scheme used in the PF-CZM decouples the displacement and damage fields, introducing an additional scalar damage variable (resulting in approximately four DOFs per node). This solution scheme enhances stability in convergence-wise demanding problems [50]. Nevertheless, the iterative nature of the staggered approach, combined with the additional degree of freedom per node required by the phase-field variable, inherently entails a significantly higher computational cost.

The computational efficiency and resource requirements for both frameworks, specifically for the models employing the maximum mesh refinement level (*h* = 0.2 mm for XFEM-CZM and *h* = 0.05 mm for PF-CZM), are summarized in Table 1. According to the data, the PF-CZM models involved approximately 7 to 8 million DOFs, requiring between 23.5 and 24.0 h of computation with a peak memory usage of 29.4 GB. In comparison, the XFEM-CZM counterparts required only 2.0 to 3.0 million DOFs, completing the simulations in 4.5 to 7.0 h with a memory footprint below 19 GB.

Despite its higher computational cost, PF-CZM provided a more robust convergence behavior, without requiring the extensive parameter tuning needed in XFEM-CZM to prevent stiffness matrix ill-conditioning. It should be noted that the high-resolution PF-CZM models reached the practical computational limits of the available workstation, highlighting the substantial computational demand inherently associated with this method.

#### 3.2.2. Fracture Behavior and Damage Evolution Analysis

**Figure 8:**
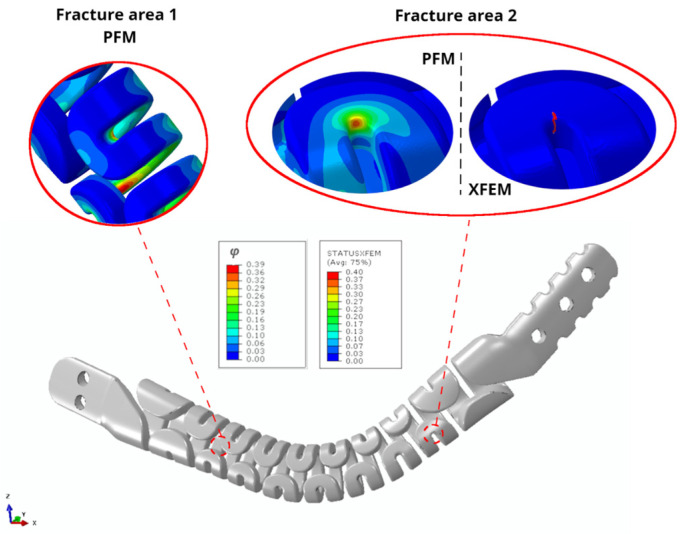
Fracture prediction in the unilateral implant model is illustrated using the damage function φ from the PF-CZM approach and the STATUS XFEM variable from the XFEM-CZM approach. The values range from 0 to 1, representing the intact and total damaged states of the material, respectively.

Figure 8 presents the fracture prediction for the unilateral implant configuration, displaying the damage function *φ* in the PF-CZM and the *STATUS XFEM* variable in the XFEM-CZM, both ranging from 0 to 1 to indicate the material damage level.

The regions exhibiting the highest levels of damage in the unilateral implant were located, first, on the curved surface of the second upper spring near the anterior end of the prosthesis (fracture area 1), and second, on the curvature of the second lower spring toward the posterior end of the implant (fracture area 2), as depicted in Figure 8. In both regions, the maximum damage value reached 0.39, indicating a relatively limited extent of damage in the fracture prediction. In contrast, the XFEM-CZM predicted crack initiation in fracture area 2 with a maximum damage variable of 0.40, coinciding with the diffuse damage zone predicted by PF-CZM in this region. However, unlike PF-CZM, which also identified damage in fracture area 1, XFEM-CZM did not predict any additional crack initiation in the material.

**Figure 9:**
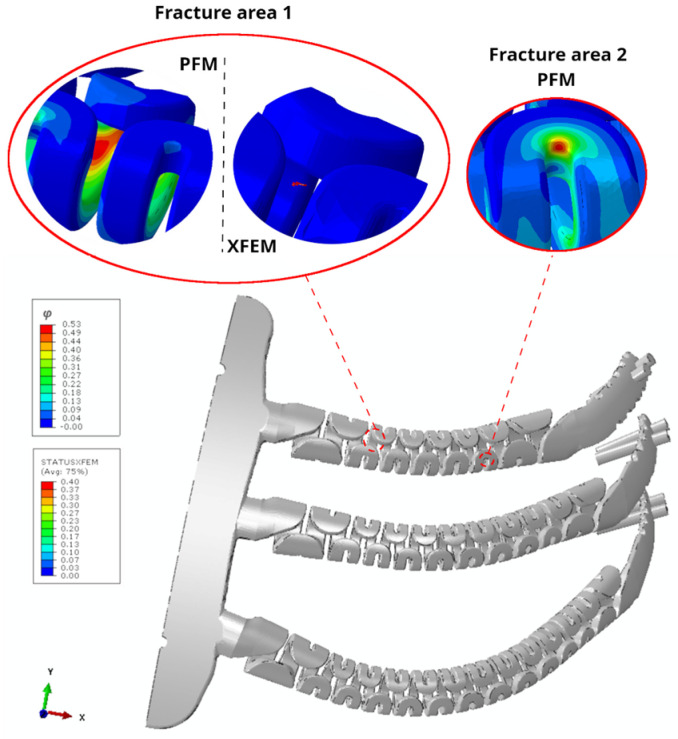
Fracture prediction in the bilateral implant model is illustrated using the damage function φ from the PF-CZM approach and the STATUS XFEM variable from the XFEM-CZM approach. The values range from 0 to 1, representing the intact and total damaged states of the material, respectively.

In the case of the bilateral implant, PF-CZM also revealed two regions of increased material damage. One was located on the first curved surface of the second upper spring, closest to the anterior end of the prosthesis, while the other was identified in the curvature of the second lower spring near the posterior end of the implant (Figure 9), similar to the observations in the unilateral model. In this case, the maximum value reached by the *φ* function was 0.53. In contrast, XFEM-CZM predicted crack initiation in fracture area 1, coinciding with the diffuse damage zone identified by PF-CZM, with a maximum damage variable of 0.40. However, as in the unilateral model, it did not predict any additional fracture initiation as proposed by PF-CZM in fracture area 2 (Figure 9). According to the results, PF-CZM revealed a broader distribution of damage regions in the implants because, as a PF formulation, it initiates damage implicitly by minimizing the total energy of the system [56,57]. This variational principle, together with the ability to regularize fracture over a finite length scale, enables the development of distributed, subcritical damage across several regions, thus revealing diffuse damage zones that may not yet satisfy an explicit initiation criterion. In contrast, the XFEM-CZM approach localizes damage more abruptly, as crack initiation requires an explicit damage initiation criterion. This criterion requires that both the local strength criterion and the corresponding energetic threshold are simultaneously exceeded, concentrating the fracture process at the most critical stress and energy concentration.

**Figure 10:**
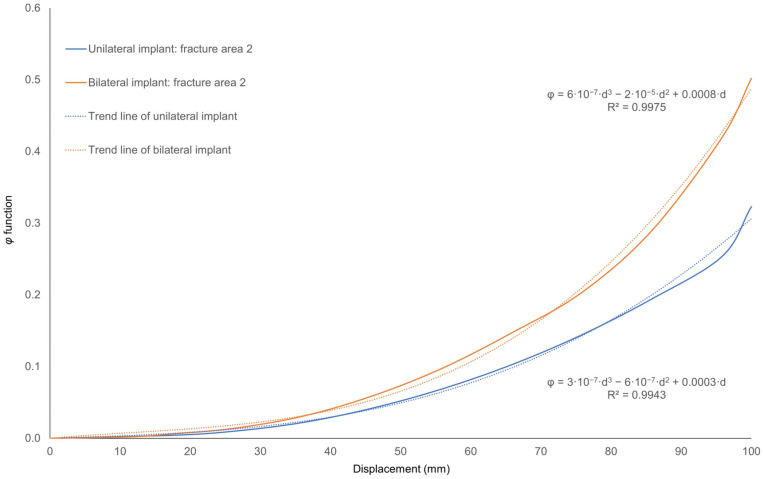
Evolution of the damage function value in fracture area 2 predicted by PF-CZM for the unilateral and bilateral implant models as a function of the applied maximum displacement with a maximum value of 100 mm. Cubic polynomial trend lines were fitted to each model to quantify the progression, where d represents the applied displacement and φ denotes the damage function.

Following the simulations, it can be observed in all cases that the material does not undergo complete fracture after the application of a maximum pure compression displacement of 100 mm to the implant, demonstrating the hyper-flexible behavior of the prosthesis. Figure 10 also depicts the evolution of the damage function *φ* within the same fracture region predicted for both models—the unilateral and bilateral implants—corresponding to fracture area 2 in each case. In each model, a cubic polynomial trend line was fitted to quantify the progression of the damage function, yielding a correlation coefficient of R = 0.99 in both cases. This indicates that although the implant was able to withstand this maximum displacement, slight increases in the applied maximum displacement could lead to permanent fracture in the regions predicted by PF-CZM.

**Figure 11:**
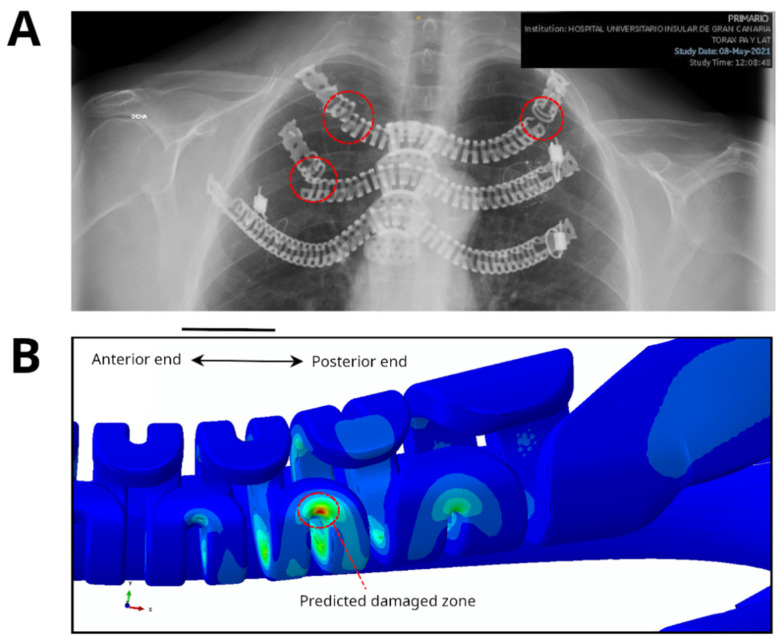
(**A**) X-ray of a fracture occurring in a clinical case reported in 2018 [20], taken on 8 May 2021, at the Hospital Universitario Insular de Gran Canaria, involving one of the earliest implant designs. (**B**) Damage region predicted by PF-CZM in the latest improved designs for both the unilateral and bilateral models.

Furthermore, fracture area 2 predicted by PF-CZM in both models was compared with the fracture pattern observed in a clinical case reported in 2018 [26], in which a bilateral implant was employed to reconstruct the first three rib levels in a patient treated with one of the earliest designs, which had not yet been optimized in the critical sections and eventually fractured (Figure 11). It is important to emphasize that this comparison cannot, under any circumstances, be considered a validation of the numerical results obtained in this work, since the reconstructed rib levels were not the same, the implant model employed in this study differed from that used in the clinical case—having been subsequently optimized in the critical sections—and the loading conditions were entirely different, as the specific loading scenario at the moment of fracture was unknown. Nevertheless, it could be valuable to compare the fracture mode observed in a previously documented clinical case with the simulations carried out in this work, as this would provide information on the fracture behavior of these flexible implant designs under different conditions. In both cases, it appears that the critical fracture regions were concentrated near the posterior ends, where abrupt geometric transitions occur, as depicted in Figure 11B. Additionally, it is noteworthy that despite the fractures observed in Figure 11A, this implant did not need to be removed, as it maintained its structural integrity and the fracture was completely asymptomatic, being detected only during a scheduled follow-up radiograph. This observation could provide further insight for future design iterations aimed at improving the biomechanical performance of the implants and ultimately achieving a longer service life.

While this was one of the earliest reported fracture cases, many subsequent thoracic reconstruction procedures using the improved implant design have been performed with satisfactory outcomes [2,26,58,59,60,61], largely due to the high flexibility achieved by the optimized design, as reflected in the simulation results in this work. This highlights the importance of performing simulations that incorporate advanced models for the design and optimization of thoracic implants. Several studies in the scientific literature have investigated quasi-static analyses of stress and strain distribution in flexible thoracic implants [8,9,10,11], but none have incorporated advanced computational models to analyze in detail the critical regions that may lead to fracture in a flexible implant, taking into account the large deformations imposed by its design.

**Figure 12:**
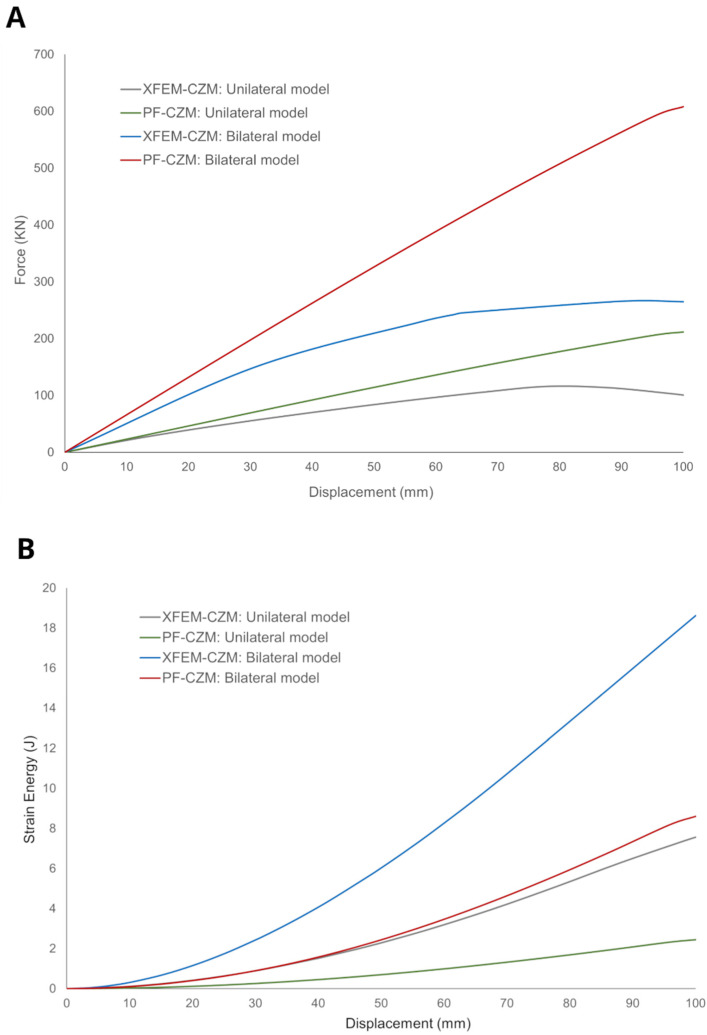
(**A**) Force–displacement curve for each implant model: unilateral and bilateral. (**B**) Elastic strain energy accumulated during the deformation process in both models.

In addition, the force–displacement curve was obtained for each model, considering both the compressive deformation and the corresponding reaction force component at the costovertebral joints (Figure 12A). For a maximum displacement of 10 cm, the bilateral model yielded a peak force (*F_c_*) of 607.95 N for PF-CZM, while XFEM-CZM reached 264.78 N. Similarly, for the unilateral implant, the maximum force was 211.49 N for PF-CZM and 100.69 N for XFEM-CZM. Moreover, the force–displacement curves exhibited a consistently increasing trend for PF-CZM in both models, whereas XFEM-CZM began to display a descending trend after 62.97 mm of displacement in the bilateral model and after 87.87 mm in the unilateral model. In addition, the strain energy stored in each of the four simulations was also computed (Figure 12B). For the bilateral model, the maximum accumulated energy was 8.60 J for PF-CZM, while for the unilateral model, the values were 2.45 J and 7.57 J, respectively. In all cases, the strain–energy curve showed a steadily increasing pattern, indicating that no permanent damage occurred in the material that would have resulted in a sudden release of the stored energy.

This divergence in peak load can be attributed to the way damage is handled by each method. In XFEM-CZM, the crack is represented as a sharp discontinuity that effectively severs the load-bearing path as soon as the cohesive elements fail, leading to the observed softening behavior after 62.97 mm of displacement in the bilateral model. In contrast, PF-CZM utilizes a regularized damage field (*φ*) that distributes the loss of stiffness over a finite volume. This diffuse damage allows the structure to continue redistributing stresses across a broader region, preventing an abrupt drop in the reaction force and resulting in a stiffer global response under the complex mixed-mode loading conditions.

The energy profiles in Figure 12B further clarify these differences. Interestingly, despite reaching lower peak forces, the XFEM-CZM models stored significantly higher levels of strain energy (up to 18.6 J in the bilateral case) compared to PF-CZM with a value of 8.6 J. This phenomenon is a direct consequence of the localization of damage. In XFEM, because the damage is strictly confined to the crack surface, the remainder of the implant volume remains fully elastic and continues to store strain energy as the displacement increases. Conversely, in PF-CZM, the damage variable degrades the elastic properties of a larger material volume (the regularization zone), effectively consuming the potential energy that would otherwise be stored as elastic strain energy.

**Figure 13:**
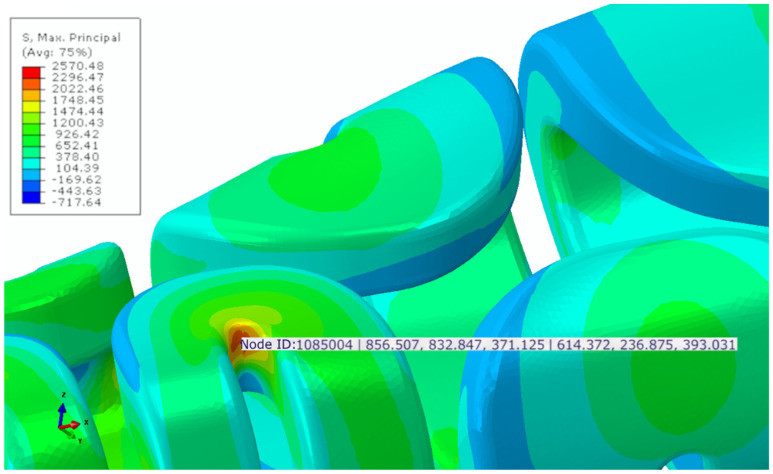
Maximum principal stresses obtained in fracture zone 2 of the unilateral model in all directions. In order: ID of the analyzed node and principal stresses S11, S22, S33, S12, S23, and S13 according to the reference coordinate system.

All of the marked discrepancies observed between PF-CZM and XFEM-CZM can be rationalized by considering the inherent assumptions of each approach. The PF-CZM provides a variational framework in which the crack path emerges naturally from the solution of the governing equations, allowing for an accurate description of fracture under mixed-mode or complex loading conditions with no extra fracture criteria needed [62]. However, it should be noted that the phase-field formulation represents fracture through a regularized damage field, which inherently leads to a distributed damage zone rather than a sharp discontinuity. Therefore, part of the observed differences with XFEM may arise from the intrinsic formulation of the method, rather than exclusively from physical differences in fracture behavior. In contrast, according to the results obtained in this study, XFEM-CZM showed similar results when the failure process was dominated by mode I and the crack trajectory remained relatively simple. Under combined and non-proportional loading, as observed in the simulated conditions (Figure 13), XFEM-CZM defined with an explicit criterion for damage initiation underestimated the contribution of shear and bending components, leading to less accurate predictions of peak forces and stored energies in the models. The magnitude of these complex stress components can be visualized in Figure 13, which highlights the multiaxial stress state in fracture zone 2 of the unilateral model. Specifically, at a critical node within this region, high values were recorded for both normal and shear components: for instance, while normal stresses reached 856.51 MPa (*S*_11_) and 832.85 MPa (*S*_22_), significant shear stresses were also present, such as *S*_12_ = 614.37 MPa and *S*_13_ = 393.03 MPa. These figures confirm that the loading is far from a simple Mode I scenario, explaining why the PF-CZM—which naturally accounts for mixed-mode energy release—provides a more consistent prediction of the overall structural response. Consequently, PF-CZM was better suited for scenarios in which the stress field was non-uniform and the crack path was not known a priori, whereas XFEM-CZM could remain advantageous in problems with dominant mode I behavior or well-defined crack propagation paths.

It should be emphasized that these observations are dependent on the modeling assumptions and numerical implementations of each approach and should not be interpreted as a general superiority of one method over the other. In particular, the applicability and performance of PF-CZM in predominantly brittle fracture problems may depend on an appropriate calibration of the regularization length scale to achieve sufficient crack localization. Furthermore, the observed limitations in XFEM may be influenced by its implementation as a ‘black box’ within commercial software, where specific convergence algorithms and crack-tracking routines are proprietary and inaccessible for adjustment. However, evaluating these tools in their commercially available form is of high clinical and engineering relevance, as they are broadly used in the medical device industry [16,18].

It is important to note that the predictive capability of XFEM-CZM could be improved by adopting more complex traction–separation laws calibrated using experimental data that specifically characterize fracture in combined-mode regimes. This highlights the importance of selecting the fracture model to the dominant failure mechanisms: while PF-CZM offers greater generality for intricate loading scenarios, a properly calibrated XFEM-CZM can remain an efficient alternative when suitable mixed-mode experimental calibration data are available.

## 4. Conclusions

PF-CZM and the standard commercial implementation of XFEM-CZM demonstrated substantial agreement in predicting fracture behavior for a relatively simple case: the fracture test of a CT specimen under plane strain conditions with a dominant mode I fracture, even when compared with real experimental data. However, when applied to more complex models of the prosthesis—both unilateral and bilateral configurations, where loading conditions were more variable—greater discrepancies were observed. These differences should be interpreted considering the distinct theoretical and numerical formulations of the PF-CZM and XFEM-CZM approaches, rather than as an intrinsic superiority of one method over the other. In these practical scenarios, PF-CZM showed a more extensive distribution of damage zone within the implants. It should be noted that this behavior is also influenced by the regularized nature of the phase-field formulation, which allows for diffuse damage representation and may facilitate the capture of distributed fracture processes. In contrast, the built-in commercial XFEM-CZM solver, configured with an explicit damage-initiation criterion and constrained by its closed algorithmic formulation, was unable to predict some fracture zones captured by PF-CZM. This limitation may be associated with the specific implementation of the XFEM method within the commercial framework and the underlying assumptions governing crack initiation and propagation. Nevertheless, the regions that XFEM-CZM was able to resolve did align in location and damage magnitude with those identified by PF-CZM.

Furthermore, within the evaluated finite element framework, the PF-CZM model exhibited superior convergence behavior across the simulations. The standard XFEM-CZM showed higher sensitivity to element size in mesh-refinement areas, making it more computationally demanding to calibrate and achieve realistic, stable fracture predictions under the same applied conditions. It must be acknowledged as a limitation of this study that the commercial XFEM solver operates largely as a ‘black box’. Many internal algorithmic choices, convergence tolerances, and stabilization parameters are pre-configured and inaccessible for user modification. Consequently, it is difficult to fully decouple the fundamental limitations of the XFEM mathematical theory from the specific proprietary constraints of the software.

Although experimental tests could not be carried out due to logistical constraints—such as material fracture-toughness characterization under standardized testing or quasi-static fracture analyses of the physical implants for direct comparison—PF-CZM enabled a highly detailed estimation of the implant’s critical damage zones. In this context, the model provides a valuable computational framework for identifying regions susceptible to failure, although linear elastic isotropic properties were assumed for both the bone and the implant material. This simplification will be addressed in future work by incorporating more sophisticated material models to further enhance the biomechanical accuracy of the simulations. Overall, this approach offers a computationally efficient basis to support and guide future improvements in implant design.

Despite the favorable hyper-flexible performance of the analyzed implant designs under the deformation conditions studied in this work, the most critical damage regions identified by PF-CZM may begin to fail earlier due to fatigue effects. Therefore, further research should focus on assessing the implants’ long-term fatigue behavior to assess whether these areas require a geometric redesign in order to maximize the implant’s service life.

## Figures and Tables

**Table 1:** Summary of mesh density, degrees of freedom, and hardware resource requirements for the XFEM-CZM and PF-CZM simulations.

Case	Model	Nodes	Elements	Total DOFs	Wall Clock (h)	Peak RAM (GB)
Unilateral	XFEM-CZM	655,740	426,982	~1,967,220	4.5	14.4
Unilateral	PF-CZM	1,958,261	1,358,459	~7,833,044	24.0	29.4
Bilateral	XFEM-CZM	978,709	603,249	~2,936,127	7.0	18.9
Bilateral	PF-CZM	1,780,729	1,143,592	~7,122,916	23.5	26.6

## Data Availability

The original contributions presented in this study are included in the article. Further inquiries can be directed to the corresponding author.
